# Simple supplementation of serum-free medium produces gametocytes of *Plasmodium falciparum* that transmit to mosquitoes

**DOI:** 10.1186/s12936-024-05094-8

**Published:** 2024-09-10

**Authors:** Sabyasachi Pradhan, Prince Chigozirim Ubiaru, Lisa Ranford-Cartwright

**Affiliations:** School of Biodiversity, One Health and Veterinary Medicine, College of Medical, Veterinary and Life Sciences, University of Glasgow, Glasgow, G12 8QQ UK

**Keywords:** *Plasmodium falciparum*, Gametocyte, AlbuMAX^™^, Serum, Culture, Transmission

## Abstract

**Background:**

Human serum is a major component of *Plasmodium falciparum* culture medium, and can be replaced with AlbuMAX^™^ II, a lipid-rich bovine serum albumin, for asexual cultures. However, gametocytes produced without serum are poorly infective to mosquitoes. Serum suffers from high cost, limited availability, and variability in quality.

**Methods:**

Several commercially-available media supplements were tested for their ability to support parasite growth and production of *P. falciparum* (3D7) gametocytes in standard RPMI1640 medium containing 0.5% AlbuMAX. The impact on asexual growth and gametocyte production with each supplement was assessed and compared to standard RPMI1640 medium containing 10% human serum, as well as to medium containing 0.5% AlbuMAX alone. The infectivity of gametocytes produced with one supplement to *Anopheles gambiae *sensu stricto was assessed by standard membrane feeding assay and measuring both prevalence of infection and oocyst intensity.

**Results:**

Supplementation of medium containing 0.5% AlbuMAX with five supplements did not affect asexual growth of *P. falciparum*, and four of the five supplements supported early gametocyte production. The supplement producing the highest number of gametocytes, ITS-X, was further investigated and was found to support the production of mature gametocytes. Infection prevalence and oocyst intensity did not differ significantly between mosquitoes given a membrane feed containing gametocytes grown in medium with 0.5% AlbuMAX + ITS-X and those grown in medium with 10% human serum. Infection prevalence and oocyst intensity was significantly higher in case of ITS–X supplementation when compared to AlbuMAX alone. Infectious gametocytes were also produced from two field clones using ITS–X supplementation.

**Conclusions:**

Serum-free medium supplemented with ITS-X was able to support the growth of gametocytes of *P. falciparum* that were as infectious to *An. gambiae* as those grown in medium with 10% serum*.* This is the first fully serum-free culture system able to produce highly infectious gametocytes, thereby removing the requirement for access to serum for transmission assays.

**Supplementary Information:**

The online version contains supplementary material available at 10.1186/s12936-024-05094-8.

## Background

Malaria remains a global burden with 249 million cases reported worldwide, with 608,000 deaths [1]. The malaria parasite *Plasmodium* requires two hosts to complete its lifecycle: a human (asexual phase) and an *Anopheles* mosquito (sexual phase). Transmission takes place when mosquitoes take up gametocytes present in the human peripheral bloodstream during blood feeding, resulting in the development of infective mosquitoes, ready to infect the human host [2].

Malaria control relies heavily on chemotherapy of infected people and vector control; the recent approval of the world’s first malaria vaccines will add to the armory [3, 4]. The current malaria control strategy of the World Health Organization (WHO) includes the use of transmission-blocking chemotherapy, where anti-malarial drugs target the transmission stages (gametocytes) in the blood and thus reduce or prevent transmission [5]. Such novel transmission-blocking drugs will be particularly necessary as endemic countries shift their policy from control to elimination of malaria. New Target Candidate Profiles (TCP) include molecules with transmission-blocking activity [6], which is usually tested by the Standard Membrane Feeding assay (SMFA) that measures mosquito infection levels following feeding of the vector on gametocytes grown in vitro [7].

The successful establishment of continuous in vitro* Plasmodium* culture of blood stages was attained in 1976 by Trager and Jensen [8]. However, variation has been noted in the growth of parasites in serum collected from different people [9–11]. An alternative to serum is AlbuMAX^™^ II, a lipid-rich bovine serum albumin [12]. The many advantages of AlbuMAX over serum include the elimination of the need for ABO compatibility between erythrocytes and serum, price and availability, ease of storage, reduced biological risk from exposure to a human-derived blood product, and improved batch-to-batch consistency. However, the major disadvantage is that parasites (gametocytes) grown in AlbuMAX cannot infect mosquitoes in the laboratory at an equivalent level to those grown in 10% human serum, and often fail to produce any infection (pers. comm.). A suitable replacement for serum would allow more consistent production of infectious gametocytes and remove the difficulty of acquiring transmission-competent human serum, but has so far proved elusive.

Very little is known about the competency of AlbuMAX in producing transmission capable *P. falciparum* gametocytes. It is reported that field isolates maintained throughout with AlbuMAX^™^ II retain their capability of sexual commitment, but the ability to infect mosquitoes was not tested [13]. It has also been reported that there is a substantial concentration difference of multiple fatty acids between serum and AlbuMAX^™^ II, and the number of Stage V gametocytes produced with AlbuMAX^™^ II can be boosted by supplementation with polyunsaturated fatty acids, to the levels achieved with serum. However, the transmission capability of those gametocytes remains unknown [14].

In this study, RPMI 1640 medium containing AlbuMAX^™^ II was supplemented with different commercially-available media supplements, and their potential to produce transmission capable gametocytes of the *P. falciparum* reference strain 3D7, as well as two recently adapted cloned lines from Mali, has been evaluated in membrane feeding experiments with *Anopheles gambiae*.

## Methods

### Media preparation

Incomplete RPMI1640 (Invitrogen, Cat No–51800–019) medium was prepared with the addition of 25 mM of HEPES (VWR, Cat No–441485H), 2 g/L of glucose (Thermo Fisher, Cat No A16828.36), 2 g/L of sodium bicarbonate (VWR, Cat No–2778.260), and 50 mg/L hypoxanthine (Sigma Aldrich, Cat No–H9636), and adjusted to pH 7.2 with 1M sodium hydroxide. Complete medium with serum (CM-S) was prepared by sterile filtering incomplete medium using a 0.22 um filter and adding 10% v/v heat-inactivated blood group AB human serum. Complete medium with AlbuMAX^™^ II (CM-A) was prepared by adding 0.5% w/v AlbuMAX^™^ II (Gibco, Cat No–11021037) to incomplete medium, and sterile filtering as before. Media supplements, provided as sterile liquid concentrates, were added to small aliquots of CM-A on the day of use, at the manufacturer’s recommended concentration (Table 1).

**Table 1:** Media supplements

Supplement (supplied concentration)	Source (catalogue number)	Composition	Concentration used
ITS-A (100X)	Gibco (51300044)	Insulin–1000 mg/L Transferrin–550 mg/L Sodium selenite–0.67 mg/L Sodium pyruvate–11000 mg/L	1X
ITS-X (100X)	Gibco (51500056)	Insulin–1000 mg/L Transferrin–550 mg/L Sodium selenite–0.67 mg/L Ethanolamine–200 mg/L	1X
ITS + 3 (100X)	Sigma aldrich (I2771)	Insulin–1000 mg/L Transferrin–550 mg/L Sodium selenite–0.5 ug/ml Linoleic acid–470 μg/ml Oleic acid–470 μg/ml BSA–50 mg/ml	1X
Linoleic acid-oleic acid-albumin (100x)	Sigma aldrich (L9655)	linoleic acid and oleic acid -2 mol/mol albumin (each)	1X
Cholesterol lipid concentrate (250X)	Gibco (12531018)	Proprietary	1X

Suppliers including catalogue numbers are given

### Parasite culture

Parasites of *P. falciparum* clone 3D7 were grown in vitro using standard protocols with slight modifications [8]. Parasites were maintained in human blood (blood group O), at 5% haematocrit in complete RPMI with serum (CM-S) at 37 ℃, under a gas mixture of 96% nitrogen, 3% carbon dioxide and 1% oxygen.

Gametocyte cultures were prepared according to standard protocols [15], with the addition of treatment with 20 U/µL heparin (Sigma Aldrich, Cat No–H3149) for 3 days from day 5 [16]. For each infectious feed, two T-25 flasks (Corning, Cat No–430372) were prepared at 5 mL volumes (5% haematocrit, 0.5% parasitaemia) set up 2 days apart, bulked up to 7.5 mL (on day 3). From day 5 the cultures were treated with heparin dissolved in the medium for 3 days to remove the asexual stages. The media in the culture flasks were changed every day until they were used for infectious feeds 15 and 17 days after setup. Two clones of recent field isolates from Mali (manuscript in preparation) were grown in vitro and gametocyte cultures were prepared as was done for 3D7.

### Impact of media supplements on parasite asexual growth

Parasite cultures, using CM-A, were set up in 6 well plates at 0.2% parasitaemia per well at 5% haematocrit (1.5 mL/well). Media were replaced every day with fresh media with or without the supplements. As well as the media supplement(s) under investigation, controls of medium with AlbuMAX only were included on each plate. The parasitaemia was measured on days 2, 4 and 6 post set-up of the experiment by removal of a small amount of blood for thin smears, which were stained with Giemsa and examined under 1000 × magnification. At least 1000 RBCs were counted per well. Early gametocytes (stage II) in the D6 cultures were counted separately to estimate potential support for sexual development. The most promising of the media supplements based on the highest gametocyte numbers after 6 days of culture was selected for infectious feeding to *Anopheles* mosquitoes.

### Impact of media supplements on parasite gametocyte production and mosquito infection

Parasite cultures were set up for gametocyte production simultaneously using CM-S (10% serum), CM-A (0.5% AlbuMAX), and CM-A + ITS-X (0.5% AlbuMAX + 1 × ITS-X) throughout the culture period. One day before the infectious feed to mosquitoes, thin smears were made and stained with Giemsa, and the numbers of Stage V mature gametocytes was evaluated by microscopy. Gametocyte production was tested in three independent replicates with batches of blood and serum from different donors used on each occasion.

*Anopheles gambiae* Kisumu strain [17] mosquitoes were offered a mixture of 15 and 17 day old gametocytes via membrane feeding according to standard protocols [15], with the final gametocytaemia in the blood meals adjusted to be in the same range of 0.7–1%. Thin smears were prepared of the infectious feed immediately before it was offered to the mosquitoes, to evaluate gametocyte densities in the blood meal by microscopy. Each infectious feed experiment included gametocytes grown in medium with serum, with AlbuMAX, and with AlbuMAX + ITS-X, and the infection experiment was repeated 3 times.

Mosquitoes were maintained at 26 °C, 80% relative humidity and given fresh glucose (10%) supplemented with 0.05% w/v 4-amino benzoic acid ad libitum. Mosquitoes were dissected ten days post blood feed to visualize the oocysts on the midguts (without staining) under 400 × magnification. Prevalence (% of mosquitoes infected) and oocyst intensity (number of oocysts in the midgut) were recorded. 20–39 mosquitoes were examined for each infectious feed.

### Statistical analysis

Gametocyte numbers (Stage V) were compared between the cultures grown with serum, AlbuMAX and AlbuMAX + ITS-X based on counting of at least 1000 erythrocytes in thin smears made 1 day before the infectious feed. The data were analysed by generalized linear modelling (GLM) in R (version 4.4.0), with gametocyte counts modelled by Poisson distribution, with fixed variables of experiment, day of culture (D14 or D16) and medium supplement (serum, AlbuMAX, Alb + ITS-X) [18]. Non-significant variables were removed by backward elimination [19], and the final minimal statistically-significant model was used to obtain estimates of gametocyte numbers and 95% confidence intervals, and to compare the numbers obtained between the three media types.

Prevalence of infection was analysed using a GLM logistic regression with the fixed variables of medium supplement type, experimental feed, and gametocyte density in the blood meal. Non-significant variables were removed by backward elimination [19] and the final minimal statistically significant model was used to obtain estimated prevalence and 95% confidence intervals, and the significance of the difference in prevalence between the media supplements.

Intensity of infection (oocyst numbers) was analysed using the R package pscl (version 1.5.9) [20], to apply negative binomial, zero-inflated and hurdle negative binomial regression models, the latter two accounting for the over-dispersion and excess zeros commonly seen in oocyst data. The maximal model contained the fixed variables of media supplement type, experimental feed, and gametocyte density, and non-significant variables were removed by backward elimination. The best fit model (NB, ZINB or Hurdle NB) was determined by comparison of AIC and accuracy of prediction of number of zero values. The final minimal statistically significant model was used to obtain estimated intensity and 95% confidence intervals, and the significance of the difference in intensity between the media supplements.

## Results

### Effect of supplements to AlbuMAX medium on parasite growth and early gametocyte production

**Fig. 1:**
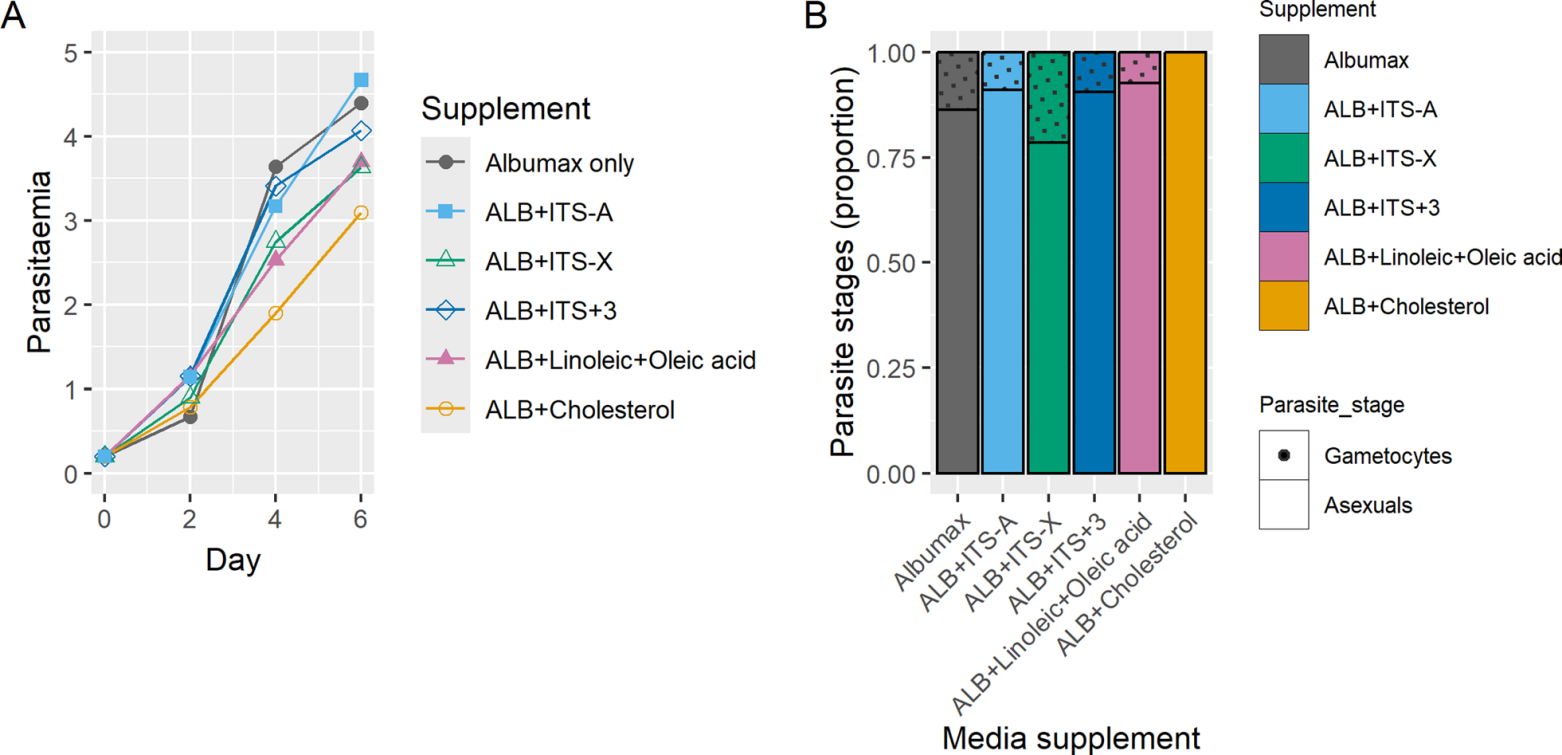
**A** Growth curve of 3D7 parasites when grown in medium containing AlbuMAX only (Control) and in AlbuMAX medium with different supplements for a period of 6 days. **B** Proportion of early stage gametocytes present on day 6 in the cultures grown in medium containing AlbuMAX only (Control) and in AlbuMAX with different media supplements

None of the five supplements tested (Table 1) adversely affected parasite growth, as measured over three asexual cycles (Fig. 1A), suggesting that these can be used in combination with AlbuMAX, at least in short term culture. Early stage gametocytes were seen on day 6 in four out of the five supplements (Fig. 1B), with the highest number seen with the supplement ITS-X. A supplement similar to ITS-X (Nutridoma-SR) has previously been shown to produce stage V gametocytes [21], and given limited availability of transmission competent serum, one supplement, ITS-X, was investigated further in mosquito transmission experiments.

### Effect of ITS-X supplementation on parasite gametocyte production and mosquito infection

**Fig. 2:**
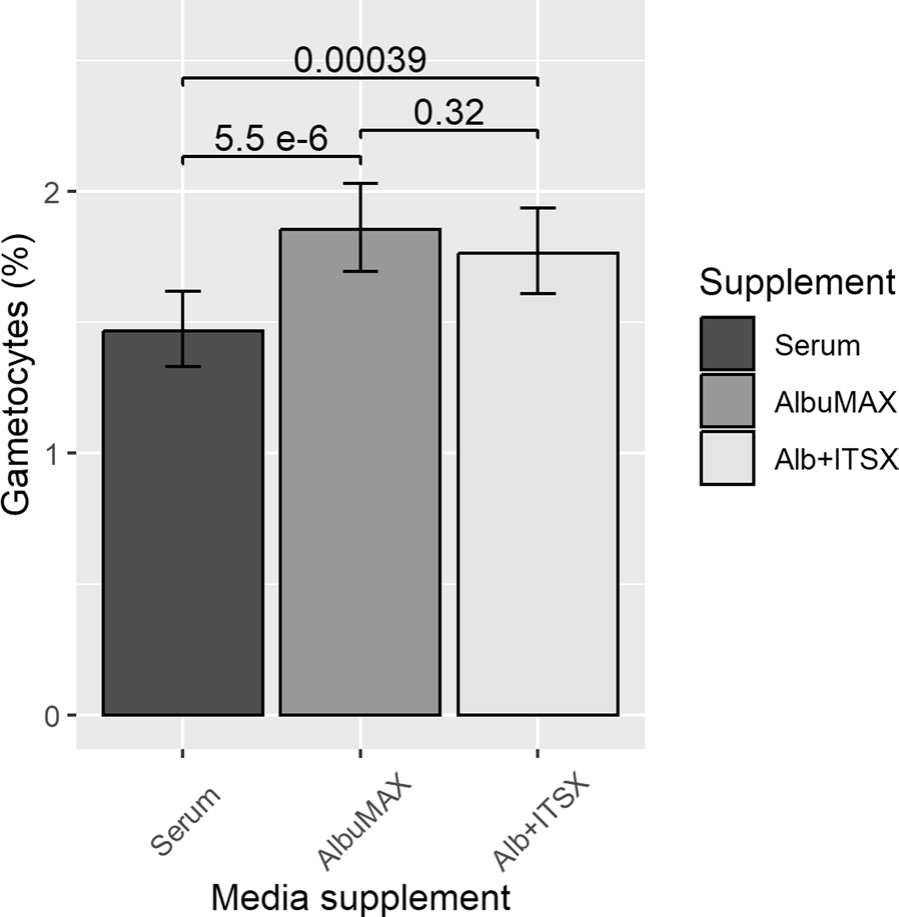
Stage V parasitaemia in cultures a day before feeding to mosquitoes. Error bars represent the 95% confidence intervals of the estimates of gametocyte numbers taken from the GLM analysis. The P values are from the GLM analyses. n = 2

Medium with AlbuMAX and AlbuMAX + ITS-X produced significantly more gametocytes than the cultures grown with 10% serum (P = 5.5 × 10^–6^ and P = 0.00039 respectively), but there was no difference in gametocyte numbers in cultures grown with AlbuMAX and with AlbuMAX + ITS-X (P = 0.32) (Fig. 2). Gametocyte numbers varied across the three independent replicates (P = 7.8 × 10^–6^), possibly because of different batches of serum.

**Fig. 3:**
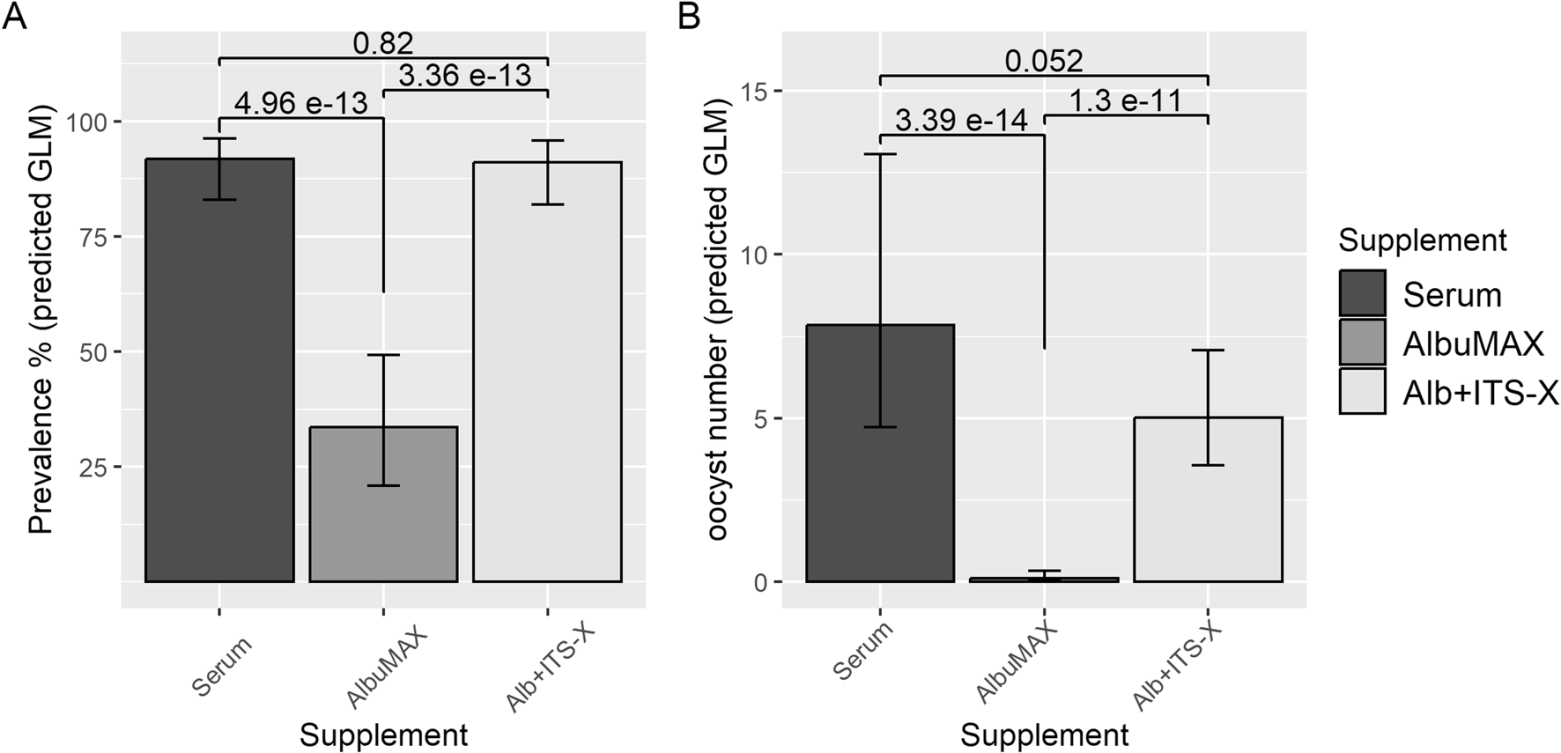
Mosquito infection using gametocytes grown in medium with 10% serum, 0.5% AlbuMAX, and 0.5% AlbuMAX + ITS-X. **A** Prevalence of infection (prediction, GLM model). **B** Oocyst intensity (predicted number of oocysts per mosquito midgut from GLM model). P values were obtained from the GLM models. Error bars represent 95% CI. n = 3

Following infectious feeding of equal numbers of gametocytes to *An. gambiae* using membrane feeding, those grown in AlbuMAX + ITS-X resulted in a mosquito prevalence equivalent to gametocytes grown in serum (P = 0.82), whereas gametocytes grown in medium with AlbuMAX alone produced significantly lower infection prevalence than serum (P = 4.96 × 10^–13^) or than AlbuMAX + ITS-X (P = 3.36 × 10^–13^), with no significant difference in gametocyte density in the infectious feed between the three medium types (P = 0.77) (Fig. 3A). The supplementation of AlbuMAX medium with ITS-X increased the infection prevalence (model predictions) from 34 to 91%.

Similar results were observed for oocyst intensity (Fig. 3B), with a similar number of oocysts predicted from the GLM models in mosquitoes given gametocytes grown in medium with serum or AlbuMAX + ITS-X (P = 0.052). Gametocytes produced in medium containing only AlbuMAX had very low predicted oocyst numbers, significantly lower than those grown in serum (P = 3.4 × 10^−14^) or AlbuMAX + ITS-X (P = 1.3 × 10^−11^).

### ITS-X supplementation of AlbuMAX^™^ II produces gametocytes from field isolates capable of establishing mosquito infection

**Fig. 4:**
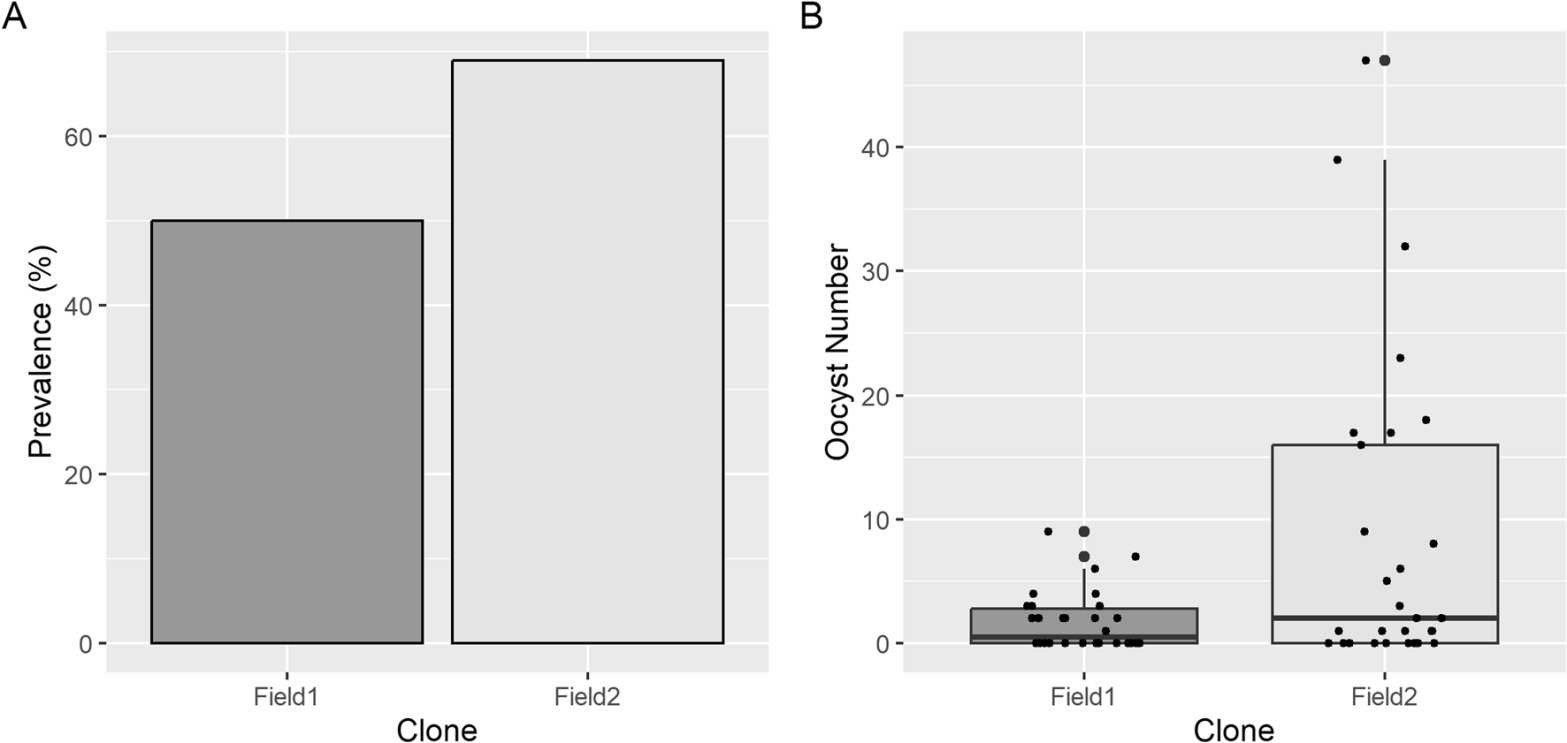
Prevalence of infection (**A**) and intensity of infection (**B**) in *An. gambiae* obtained from two field clones cultured using AlbuMAX^™^ II medium supplemented with ITS-X. Each dot is representative of an individual midgut

Gametocytes of two parasite clones made from recent field isolates (Field1 and Field2) produced using AlbuMAX + ITS-X medium were able to establish infection in the mosquito midgut with 50 and 69% prevalence (Fig. 4A) and a median oocyst intensity of 0.5 and 2, respectively (Fig. 4B).

## Discussion

Infectious gametocytes of *P. falciparum* grown in serum-free media with AlbuMAX are poorly infectious to *Anopheles* mosquitoes compared to those grown in human serum, with significantly lower prevalence and intensity of infection. The key finding of this research is that supplementation of the AlbuMAX-containing media with the supplement ITS-X resulted in increased mosquito infection prevalence and intensity, to statistically similar levels as those obtained with gametocytes grown in 10% human serum. Therefore, ITS-X supplementation of AlbuMAX-containing media provides a complete replacement for serum-containing medium for the production of fully transmission-competent gametocytes.

None of the five supplements used in the initial screen were found to be toxic to the growth of the parasite. Due to limited supplies of transmission-competent serum, only one supplement (ITS-X) was taken forward for mosquito infection studies, and the ability of additional supplements to support development of transmission-competent gametocytes was not assessed further. The number of mature Stage V gametocytes produced with ITS-X supplementation was not statistically different to those produced with AlbuMAX alone in the medium, so the increased infection rates are not due to higher numbers of gametocytes. This contrasts with the findings of a study where various combinations of polyunsaturated fatty acids was added to AlbuMAX cultures to boost production of Stage V gametocytes levels similar to that attained with serum [14], but this difference can probably be attributed to batch variation in the serum [9].

ITS-X (100X) is a commercial media supplement containing insulin, transferrin, sodium selenite and ethanolamine. Insulin, transferrin and selenium have previously been reported to promote the asexual growth of *P. falciparum *in vitro [22–24] but the most likely component of the supplement in producing transmission-competent gametocytes is ethanolamine, a key component of phospholipids in biological membranes. The lipid content and profile of gametocytes is substantially different to the asexual stages of the parasite [25–27]. Expression of several genes involved in lipid metabolism is also increased during gametocyte development [28, 29]. Ethanolamine is obtained by the parasite from the external environment and is converted to phosphatidylethanolamine (PE) via the parasite’s de novo Kennedy pathway [30, 31]. The other major phospholipid in malaria parasite membranes, phosphatidylcholine (PC), can also be produced from ethanolamine by an alternative pathway in the parasite, involving phosphatidylethanolamine N-methyltransferase (PMT) [32].

The cost and availability of quality human serum are critical limiting factors for culturing infectious gametocytes, in addition to issues such as batch-to-batch variability and the requirement for ABO blood group matching, and, in malaria-endemic countries, the presence of transmission blocking/anti-malarial antibodies in locally-acquired serum. The use of a commercially-available media supplement with AlbuMAX^™^ II removes the reliance on human serum for transmission studies including transmission-blocking assays.

In summary, sexual stage parasites grown with AlbuMAX + ITS-X medium successfully established infection in the mosquito midgut, with comparable infection prevalence and intensity to that attained with gametocytes grown with serum, both for a laboratory parasite line and two field clones of *P. falciparum*. Further studies are necessary to understand the role of the supplement components in achieving transmission.

## Conclusions

This study reports for the first time that *P. falciparum* gametocytes produced in the complete absence of serum are fully transmission-competent. The use of a commercial media supplement will reduce reliance on human serum, and thus permit more affordable, robust and reliable parasite transmission studies.

## Supplementary Information

Additional file 1

## Data Availability

The data supporting the conclusions of this article are included within the article. No datasets were generated or analysed during the current study.

## Abbreviations

AIC: Akaike Information Criterion
RPMI: Roswell Park Memorial Institute
SMFA: Standardized membrane feeding assay
TCP: Target candidate profile
RBC: Red blood cell
GLM: General linearized model
NB: Negative binomial
ZINB: Zero inflated negative binomial
PE: Phosphatidylethanolamine
PC: Phosphatidylcholine
PMT: Phosphatidylethanolamine methyltransferase
