# Supplementary material for: Simple supplementation of serum-free medium produces gametocytes of Plasmodium falciparum that transmit to mosquitoes
Source: Malar J. 2024 Sep 10;23:275. doi: 10.1186/s12936-024-05094-8 (PMC11389287; doi:10.1186/s12936-024-05094-8)
Supplement: Supplementary file 1 — Additional file 1 [file 12936_2024_5094_MOESM1_ESM.pdf]

Supplementary information

A

Treatment	Replicate	Gametocyte Density (%)	Prevalence (infected/dissected) (%)	Oocyst Range
Serum	1	1.01	22/28 (78.6%)	0-260
	2	0.69	16/20 (80%)	0-160
	3	0.92	28/31 (90.3%)	0-25
Albumax	1	1.14	2/35 (5.7%)	0-2
	2	0.94	6/20 (30%)	0-2
	3	0.77	10/30 (33.3%)	0-2
Albumax + ITS-X	1	1.91	28/39 (71.8%)	0-24
	2	0.62	16/20 (80%)	0-20
	3	0.72	27/29 (93.1%)	0-45

B

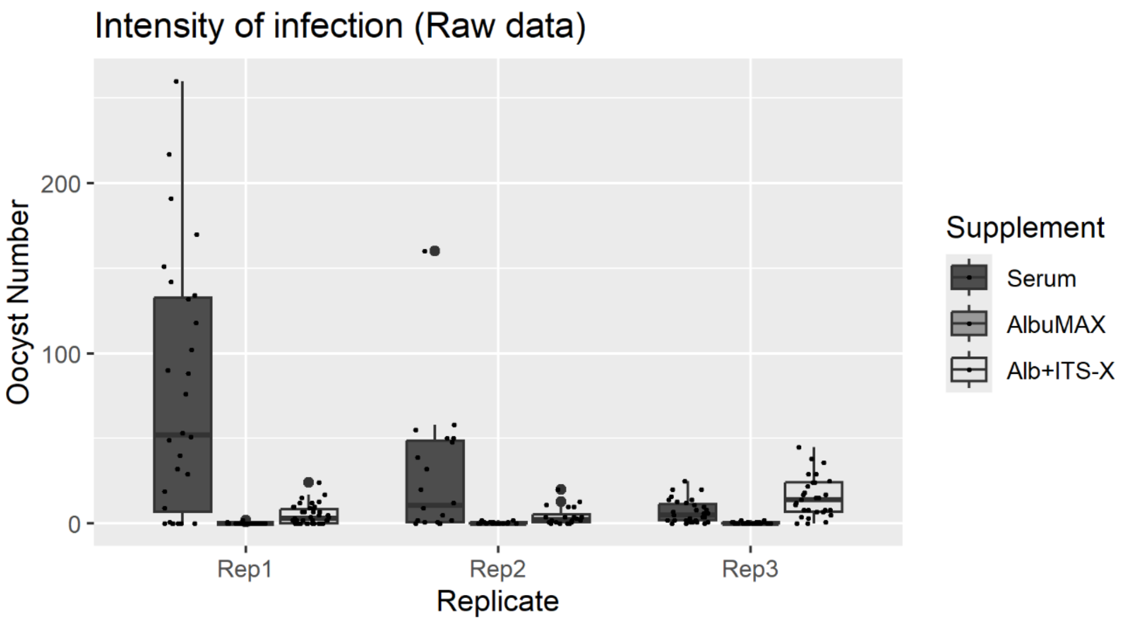

Supplementary Figure 1. (A) Table showing the raw data for the three replicate experiments performed. (B) A box plot showing the number of oocysts per mosquito dissected for the three replicate experiments. Each dot represents a single mosquito.
